# Complex Tumor Spheroid Formation and One-Step Cancer-Associated Fibroblasts Purification from Hepatocellular Carcinoma Tissue Promoted by Inorganic Surface Topography

**DOI:** 10.3390/nano11123233

**Published:** 2021-11-28

**Authors:** Francesco Dituri, Matteo Centonze, Erwin J. W. Berenschot, Niels R. Tas, Arturo Susarrey-Arce, Silke Krol

**Affiliations:** 1 Laboratory for Personalized Medicine, National Institute of Gastroenterology, “S. de Bellis” Research Hospital, Castellana Grotte Via Turi 27, 70013 Bari, Italy; Francesco.dituri@irccsdebellis.it (F.D.); matteo.centonze@irccsdebellis.it (M.C.); 2 Mesoscale Chemical Systems, MESA+ Institute, University of Twente, P.O. Box 217, 7500 AE Enschede, The Netherlands; j.w.berenschot@utwente.nl (E.J.W.B.); n.r.tas@utwente.nl (N.R.T.)

**Keywords:** hepatocellular carcinoma, cancer-associated fibroblast, tumor spheroids, structured surface, cell culture, in vitro tumor models

## Abstract

In vitro cell models play important roles as testbeds for toxicity studies, drug development, or as replacements in animal experiments. In particular, complex tumor models such as hepatocellular carcinoma (HCC) are needed to predict drug efficacy and facilitate translation into clinical practice. In this work, topographical features of amorphous silicon dioxide (SiO_2_) are fabricated and tested for cell culture of primary HCC cells and cell lines. The topographies vary from pyramids to octahedrons to structures named fractals, with increased hierarchy and organized in periodic arrays (square or Hexagonal). The pyramids were found to promote complex 2D/3D tissue formation from primary HCC cells. It was found that the 2D layer was mainly composed of cancer-associated fibroblasts (CAFs), while the 3D spheroids were composed of tumor cells enwrapped by a CAF layer. Compared with conventional protocols for 3D cultures, this novel approach mimics the 2D/3D complexity of the original tumor by invading CAFs and a microtumor. Topographies such as octahedrons and fractals exclude tumor cells and allow one-step isolation of CAFs even directly from tumor tissue of patients as the CAFs migrate into the structured substrate. Cell lines form spheroids within a short time. The presented inorganic topographical surfaces stimulate complex spheroid formation while avoiding additional biological scaffolds and allowing direct visualization on the substrate.

## 1. Introduction

Solid cancers often arise as complex biological systems composed of multiple cellular and non-cellular components that operate in highly interactive ways. Cancer-associated fibroblasts (CAFs) are gaining increasing interest due to their extensive involvement in tumor maintenance, proliferation, progression, metastasis, and serving as potential targets for interruptive tumor therapy [1]. CAFs are part of the tumor microenvironment (TME), which is represented by both non-tumor cells (including fibroblasts, endothelial, and immune cells of the innate and adaptive immunities), and non-cellular components such as growth factors, cytokines, chemokines, and extracellular matrix (ECM) molecules. Among non-cellular components, ECM is subject to active remodeling by adjacent cells, resulting in enhanced activation and motility of tumor-modified macrophages, transformed cells, pericytes, and fibroblasts, as a prerequisite for achieving neoplastic progression [2]. Furthermore, cancer supportive feature of CAFs can also be related to the excessive release of ECM described in hepatocellular carcinoma (HCC), pancreatic ductal adenocarcinoma, and other desmoplastic tumors, such as cholangiocarcinoma [3,4,5]. CAFs play crucial roles in tumor models. In fact, the depletion of CAF cells in experimentally generated tumors might result in an increased number of tumor-promoting regulatory T cells (Tregs), enhanced hypoxia, vascularity, and, ultimately, aggressiveness [6,7,8,9,10]. CAFs can also promote stemness properties of cancer cells, a condition that is likely to be related to acquired chemoresistance and failure of treatments. More specifically, chemoresistance is mediated by the release of chemokines (IL-6), metalloproteinases (MMP-9), and an excessive amount of ECM upon exposure to TGF- β [11,12,13]. Additionally, CAFs are the major communicator between tumor and its microenvironment, therefore driving tumor cell proliferation, apoptosis, migration, invasion, angiogenesis, immune escape, and drug resistance [5]. CAFs are typically identified by their expression of alpha-smooth muscle actin (α-SMA) while otherwise lacking common biomarkers due to their heterogeneous origin [5]. The detection of this biomarker is clinically relevant, as its expression is positively correlated to poor prognosis in HCC [14,15,16].

Mimicking tumor tissue complexity in in vitro models serves in better understanding biological functions, specific pathways, and reliable drug screening and toxicity studies [1]. However, cell sheets of mono-cultured tumor cell lines or even co-cultured with CAFs lack the complexity and heterogeneity of native tumors. In order to predict drug efficacy or understand complex biological interactions, complex in vitro models are necessary [17]. That spheroids/organoids or 3D co-cultures of different cell lines reflect better the in vivo situation is nowadays undoubted [18]. Consequently, the results of drug discovery in 2D often fail to translate into human therapy [19]. Therefore, 3D cell culture as tissue models is increasingly acknowledged as systems that recapitulate cellular and molecular dynamics underlying physiological and pathological processes more accurately than conventional cell sheets known as 2D cultures. More complex 3D tissue known as organoid can contain mixed single cells of disintegrated primary tissue or even tumor pieces. These patient-derived organoids (PDOs) or patient-derived xenoplants (PDXs), e.g., for liver [20,21], are then used for better understanding the disease by implanting them in immune-deficient mice. This is a process that needs several months just for growing the microtumor. Another approach uses these PDO/PDX encapsulated in an amorphous fiber, grown just for 7 days, to identify the optimal treatment for this particular patient and hence provide personalized therapy [22].

Strategies to produce 3D cell clusters can be divided into scaffold or scaffold-free. Scaffold approaches offer physical support in the form of matrices made of natural or synthetic materials suitable for optimal cell growth, cell differentiation, and cell function. Hydrogels that aid in providing nutrients and tissue support [23,24,25] are mainly used as scaffolds; this includes hydrogels or inks used for 3D printing [26,27]. Known drawbacks associated with hydrogels commonly arise from lot-to-lot variability, especially for animal-derived hydrogels [28,29]. An additional drawback is that the hydrogel may interact with secreted molecules limiting the diffusion of nutrients, or added drugs, creating concentration inhomogeneity. Other scaffolds are decellularized explants, collagen gels, synthetic polymer membranes, microfiber meshes, and many others depending on their future application [30]. For scaffolds, pore distribution, exposed surface area, and porosity are of crucial importance. The pores influence cell penetration into the scaffold and the architecture of the produced extracellular matrix [30]. 

Scaffold-free approaches induce spheroid formation by preventing cell attachment to surfaces (hanging drop, stirring, cell repellent surface, concave plate) [31,32,33,34,35,36,37,38,39]. Low attachment can be achieved by repellent coating a planar surface or incorporating micro(nano)structuring to [31,32,33,34,35,36,37,38,40]. 

To date, the use of micro(nano) topographies for 3D cell cluster formation is limited. Most of the examples for topographical surfaces can be found in literature applied to stem cell differentiation [38]. 

This work reports the use of periodically organized micro(nano)engineered structures, which chemical composition is SiO_2_, silicon dioxide, for cell culture. We studied their influence on the growth of cancer-associated fibroblast isolated from hepatocellular carcinoma (HCC) samples of patients. The structures varied in morphology, i.e., from pyramids, octahedrons to fractals of increased hierarchy organized in periodic arrays enabling surface topography. The topographies are arrayed, adopting a square (*Sqr*) or hexagonal (*Hex*) configuration. Depending on the topography, the cells grow in sheets or attached spheroids is observed. We studied the reason for the different cellular responses to the topographies in detail. A comparison was made with (i) the HCC cell line, HLF, and (ii) with cell culture in MatriGel, the standard protocol for spheroid growth. Finally, we showed some applications of the different structures, such as one-step CAF isolation. Usually, the purification of CAFs isolated from primary tumors is time-consuming, ranging from 1 day up to 80 days to achieve a pure CAF culture [41,42,43,44].

## 2. Materials and Methods

### 2.1. HCC Tissue

This study falls under the approval given by the local ethics committee, Azienda Ospedaliero Universitaria Consorziale Policlinico di Bari (Bari, Italy); protocol number: 254; date of release: February 2012.

Immediately after surgical resection, hepatocellular carcinoma (HCC) tissue and peri-tumoral (non-tumor tissue) specimens were cut into 0.5–1 cm pieces and left in MACS tissue storage solution (Miltenyi Biotec, Bergisch Gladbach, Germany). These tissue fragments were cut into smaller size pieces (1–2 mm). Then, the HCC and peri-tumoral tissue pieces were either planted directly on the templates or isolated to enhance the number of cancer-associated fibroblasts. For the study, several CAF isolates from different patients were used.

### 2.2. CAF Isolation

The 1–2 mm tissue pieces were washed three times in Hanks balanced salt solution (HBSS) and then incubated in HBSS in the presence of collagenase Type IV (Thermo Fisher Scientific, Waltham, MA, USA) and 3 mM CaCl_2_ at 37 °C under gentle rotation for 4 h. At the end of this step, the dissociation was mechanically facilitated by pipetting up–down the digested tissues with a large size orifice 50 mL pipet. The floating cells were collected and washed three times with HBSS and kept in this solution on ice (1st digestion round). The decanted partially digested tissue specimens were subjected to the second round of digestion (as described above). The resulting dissociated cells (2nd digestion round) were washed twice with HBSS, then combined with cells from 1st digestion round, and centrifuged at 80× *g* for 5 min to separate epithelial and fibroblast cells. The fibroblasts in the supernatant were centrifuged at 100× *g* for 10 min. According to the manufacturer’s instructions, the fibroblasts in the pellet were purified through positive selection using anti-fibroblasts MicroBeads MS Column (Miltenyi Biotech, Bergisch Gladbach, Germany). CAFs were then cultured in improved minimum essential medium (IMDM), a modified Dulbecco’s modified Eagle medium (DMEM) with 20% fetal bovine serum (FBS, Thermo Fisher Scientific, Waltham, MA, USA). In traditional cell culture, repeated passage and long-term culture in a selective medium are used to purify CAFs in 2D standard cell culture [41]. 

In our study, CAFs isolates were used for cell culture experiments under standard culture conditions. The isolate from primary CAFs preparation was grown for 8 and 13 days, then fixed for 10 min with 4% paraformaldehyde in phosphate-buffered saline (PBS) at pH = 7.4 and then treated for immunohistochemistry. For each of these experiments, the starting cell density was 2 × 10^4^ cells/mL. This heterogeneous cell mixture was cultured on the sterilized substrates (1 cm × 1 cm) containing pyramids (G0) and octahedrons (G1).

### 2.3. HLF Cells

HLF (JCRB Cell Bank, JCRB0405, Osaka, Japan) is a non-differentiated HCC cell line. The cells were cultured in DMEM medium (Gibco), supplemented with 10% FBS, 1 mM pyruvate, 25 mM HEPES, 100 U/mL penicillin–streptomycin, and maintained at 37 °C an atmosphere containing 5% CO_2_. The 2D cultured cells were trypsinized and resuspended in a complete DMEM medium at 4 × 10^5^ cells/mL concentration. Then, 50 µL of cell suspension (containing 2 × 10^4^ cells) was seeded on the sterile substrates. First, the single cells were incubated for 4 h at 37 °C and 5% CO_2_ without an additional medium in, to allow them to attach exclusively onto the 1 cm × 1 cm topographical surface containing pyramids and octahedrons. Then, the substrates were covered with 3 mL of complete medium and placed in the incubator, refreshing the medium every 3 days.

### 2.4. Cell Culture on Topographical Surfaces 

In all cases, the substrates with the topographical surfaces were placed in 6-well plates for experiments in triplicates or in 24-well plates if only one template was used. Before cell seeding, the well plates containing substrates were sterilized by irradiation with UV light in the laminar flow hood for 1 h.

Cells were trypsinized and resuspended in a medium at the concentration of 4 × 10^5^ cells/mL. Briefly, 50 μL of cell suspension (containing 2 × 10^4^ cells) were seeded on the sterilized substrates. First, the cells were incubated for at least 4 h at 37 °C and 5% CO2 without an additional medium, allowing them to attach to the substrate. The pre-incubation leads to an inhomogeneous distribution of the cells on the surface, as can be seen in the overview image depicted in Figure S1 in Supplementary Materials: the image is merged from 12 images using the FIJI stitching plugin [45].

Then, the substrates were covered with an additional medium and placed in the incubator, changing the medium every 3 days. On day 8 and day 13, one substrate for each sample was fixed for 10 min with 4% paraformaldehyde in phosphate-buffered saline (PBS) at pH = 7.4. Each experiment was performed in duplicate or triplicate.

### 2.5. Imaging of Cells on Topographical Surfaces

For the fluorescence imaging of actin filaments, the fixed cells were permeabilized with 0.1% Triton X-100 in PBS (2% bovine serum albumin added) for 15 min and then incubated for 1–2 h in the presence of phalloidin–tetramethylrhodamine B isothiocyanate (TRITC; Sigma-Aldrich, Darmstadt, Germany) to visualize the actin cytoskeleton. To distinguish CAFs from tumor cells, the cells were stained with anti- α-fetoprotein (AFP) antibodies covalently bound to Alexa Fluor488 (tumor) and for α-smooth muscle actin (α-SMA; CAF). Detection of α-SMA and α-fetoprotein expression by immunofluorescence imaging was performed on 4% paraformaldehyde-fixed cells. Fixed cells were permeabilized with 0.1% Triton X-100 in PBS for 10 min. Cells were washed three times with PBS and then incubated with 1% BSA in PBS (PBS+ 0.1% Tween 20) for 30 min to block unspecific binding of the antibodies and thereafter incubated with the diluted antibodies in 1% BSA in PBS overnight at 4 °C (α-SMA: Cell Signaling Technology, 1:100; AFP: BD Pharmingen, 1:100). The cells were washed three times in PBS, and for α-SMA, they were incubated with a secondary Antibody Alexa Fluor^®^ 488 conjugate (Invitrogen) diluted in 1% BSA in PBS (1:50) for 1 h at room temperature in the dark. 

All cells were washed three times with PBS after staining. Then, the adhered cells were covered with 4′,6-diamidino-2-phenylindole (DAPI)-supplemented antifade mounting medium VECTASHIELD (Vector Lab, Burlingame, CA, USA) to stain the nucleus, followed by immediate visualization in fluorescence microscopy (NIKON Eclipse Ti2, Japan; 10× objective). No additional treatment to the topographical surface was needed for imaging since the surface is compatible with most light microscopy techniques.

For each sample, 3–10 images were recorded in different positions on the template. 

### 2.6. Flow Cytometry

The composition of the CAFs isolated from biopsies of primary tumor nodules explanted from three HCC patients was characterized by flow cytometry. Analysis of markers to detect HCC cancer cells and CAFs was performed using the following anti-human antibodies: Alexa Fluor 488-conjugated IgG2a to alpha-fetoprotein (AFP, BD Biosciences, San Jose, CA, USA); FITC-conjugated IgG1 to CD13 (Merck, Germany); FITC-conjugated IgG2b to CD44 (BD Biosciences, USA); FITC-conjugated IgG1 to CD90 (BD Biosciences, USA); FITC-conjugated IgG1 to CD133 (Miltenyi Biotec, Bergisch Gladbach, Germany); Unconjugated IgG1 to CD151 (abcam, Cambridge, UK); FITC-conjugated IgG2b to EpCAM (BioLegend, San Diego, CA, USA); Unconjugated IgG1 to OV-6 (R&D Systems, Minneapolis, MN, USA); FITC-conjugated IgG1, IgG2a and IgG2b isotype control antibodies (Miltenyi Biotec, Bergisch Gladbach, Germany); Alexa Fluor 488-conjugated IgG isotype control antibody (abcam, Cambridge, UK); Alexa Fluor 488-conjugated anti-mouse antibody.

Briefly, the cells were detached using StemPro Accutase Cell Dissociation Reagent (Thermo Fisher Scientific, Waltham, MA, USA) and incubated with fluorophore-conjugated antibodies for surface staining of CD13, CD44, CD90, CD133, CD151, EpCAM, and OV-6 for 1 h at 4 °C in the dark. For AFP staining, cells were fixed and permeabilized using Foxp3/transcription factor fixation/permeabilization concentrate and diluent (eBioscience, Thermo Fisher Scientific, Waltham, MA, USA), prior to antibodies incubation. A second incubation step with a secondary Alexa Fluor 488-conjugated antibody (for 1 h at 4 °C in the dark) was performed to detect CD151 and OV-6. Fluorophore-conjugated isotype antibodies were used as controls related to the detection of AFP, CD13, CD44, CD90, CD133, EpCAM. Alexa Fluor 488-conjugated anti-mouse antibody was used as a control related to the detection of CD151 and OV-6. Cells were analyzed using the Navios flow cytometer, and the data were processed using the Kaluza Software 1.5 (Beckman Coulter, Brea, CA, USA).

## 3. Results

### 3.1. Topographical Surfaces

**Figure 1:**
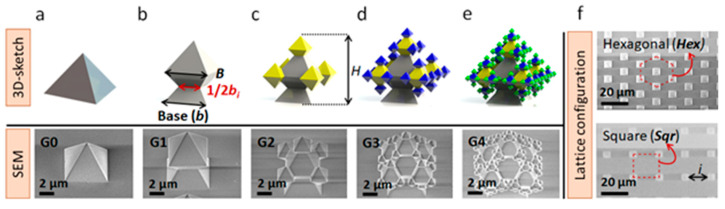
A 3D sketch of the various fabricated structure geometries (**a**–**e**). Each sketch is linked to an SEM image in the display below. The structure geometries increased in self-similarity and complexity (**a**) G0, (**b**) G1, (**c**) G2, (**d**) G3, and (**e**) G4. Panel (**f**) highlights the two lattice configurations.

The 3D structures and the underlying surface of the topographies shown in Figure 1 consist of SiO_2_. The structures were divided into (i) topographies with low hierarchy named pyramids and octahedrons, as shown in Figure 1a,b, and (ii) topographies with high hierarchy named fractals, as shown in Figure 1c–e. Fractal structures had self-similar units, as highlighted by the colored features in Figure 1c–e. Each structure had different variations between each geometrical (G) feature in Figure 1. SEM image of the fabricated topography is displayed below the fabricated structures in Figure 1a–e and labeled G0 to G4 as the geometry complexity increases. Each fabricated surface had a particular lattice configuration that varied from a hexagonal (*Hex*) to a square lattice (*Sqr*), as presented in Figure 1f.

From SEM images in Figure 1, structure hierarchy is demonstrated. Starting from G1, it is evident the presence of octahedral units could expand five times more than the previous generation, with half of the base increase in size (1/2b_i_ = 1, 2, 3, 4) (e.g., Figure 1a,c) reaching a fractal dimension unit of 2.322 [46]. However, a non-random quasi-exact fractal size distribution could be seen over the smallest and largest octahedral features (Figure S2). Interestingly, relatively high surface-area-to-volume ratios between 1.1 and 1.6 per fractal unit in Figure 1b–e were obtained [46,47]. These units could then be expanded over larger areas forming arrays of defined interspaces (i) and heights (h) over different lattice configurations. It should be noted that parameters such as b, 1/2b, and H remained similar between *Sqr* and *Hex* lattice configurations and are summarized in Table S1. More details about the fractal dimensions can be found in Figure S2. Details on the fabrication process of the topographies in Figure 1 are presented in the supporting information, which can be found in the section named fabrication of the topographical surfaces. A similar fabrication process as described in [46,47] was followed for the planar substrates used as controls. In short, planar substrates involved the growth of SiO_2_ over a silicon wafer, which was then bonded to the glass. After bonding, the silicon was back etched, leaving the same type of SiO_2_ used to produce the 3D structures from Figure 1 over the glass. The planar surfaces were used as control samples during cell growth experiments.

### 3.2. CAF Response to Topographic Surfaces of Increasing Complexity

**Figure 2:**
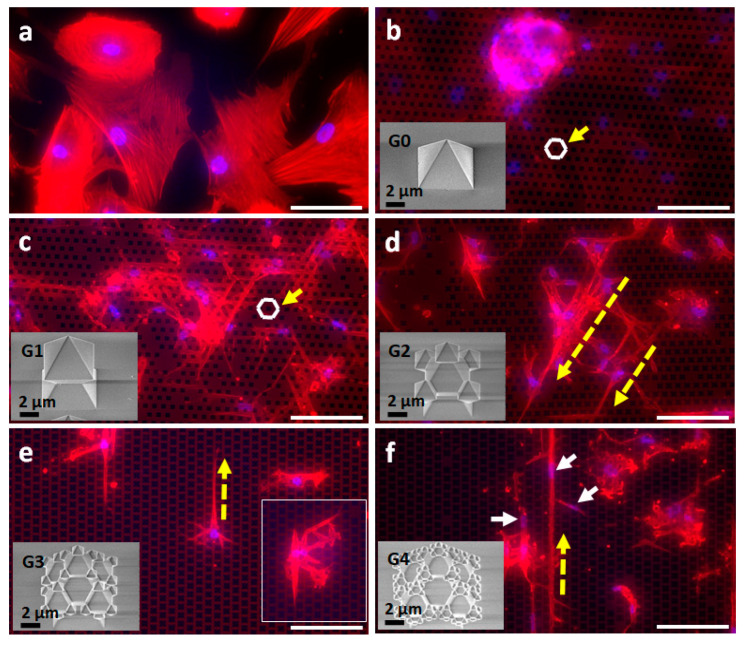
Epifluorescence images of CAFs 13 days after seeding on a *Hex*agonal lattice of SiO_2_ fractal surfaces: (**a**) control: flat SiO_2_; (**b**) G0*Hex*; (**c**) G1*Hex*; (**d**) G2*Hex*; (**e**) G3*Hex*; (**f**) G4*Hex*. The nucleus was stained by DAPI (blue), while actin filaments were visualized by TRITC–phalloidin (red). The underlying structures were visualized by transmission light. White arrows in (**f**) indicate elongated nuclei. The yellow arrows along with the white *Hex*agon in (**a**,**b**) highlight the *Hex* lattice configuration. Dashed yellow arrows in (**d**,**e**) are used to emphasize the cell directionality. Scale bar: 100 μm.

First, CAFs isolated from HCC patients were seeded on the topographic surfaces with different topographies and orientations (Figure 2 and Figure S3), and their growth behavior was monitored for up to 13 days by light microscopy. On day 8 and day 13, the cells were fixed and fluorescently stained with DAPI to visualize the nucleus (blue) and by TRITC–phalloidin for the actin filaments of the cytoskeleton (red).

On the planar SiO_2_ surface, the morphology was as expected in 2D cell culture. Cells showed extended areas of stress fibers (Figure 2a or Figure S4a). In contrast, on the pyramidal G0 for both orientations (Figure 2b or Figure S4b), we observed 3D cell clusters with a diameter of 100 to 200 μm formed on underlying CAFs. The spheroids stemmed from loosely connected cell clusters, which formed already on day 1 and grew into large more compact spheroids within 6 days (Figure S3). In general, no significant differences in cell growth were found between the two orientations.

**Figure 3:**
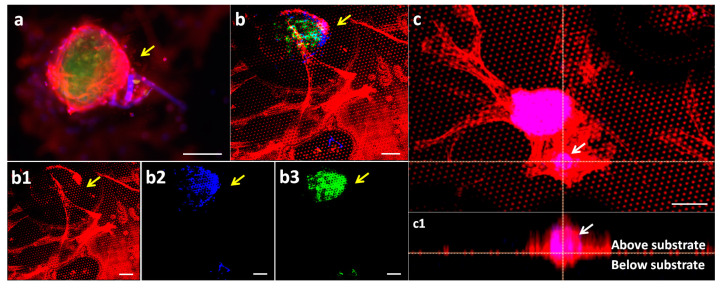
Spheroids grown on G0*Hex* surface: (**a**) confocal image of the spheroid. Tumor cells are positive for AFP (green) enwrapped by CAFs positive for α-SMA (red) (see yellow arrow); (**b**) merged fluorescence image of the red channel for CAF, blue channel for nuclei, and green channel for tumor cells. (**b1**–**b3**) separate fluorescence channels show the 2D cell layer consists of CAFs and connects the tumor with the cell layer. DAPI stains the nucleus (blue); (**c**) Z-stack of a confocal micrograph of two spheroids (x-y view) on an α-SMA (red) positive CAF layer. The signal of DAPI in the CAF layer is very dim because the SMA signal is so strong. However, due to co-localization of DAPI (blue) with the SMA signal (red) the spheroid/microtumors appear pink; (**c1**) shows the x-z view. Scale bar: 100 μm.

As the hierarchy of the surface topography increased (G1–G4), only a 2D cell layer was observed. CAFs in Figure 2c–f appear as spindle-like cells with elongated nuclei (white arrows in Figure 2f, Figure 3c1, or Figure S5) and well-developed lamellipodia connected to the fractal structures (inset Figure 2e). Directionality and elongated cytoskeleton (yellow dashed arrows in Figure 2d–f) could be attributed to the surface topography. The cell nuclei were mainly located between the structures. Lamellipodia interacted with the structures, as indicated by the high concentration in actin (red signal in Figure S5a). A detailed study on the influence of the more complex surface topographies (G1–G4) on cells was not carried out for this manuscript. Morphology, proliferation, proteomics, and genomics of primary CAFs on these surfaces are planed in future studies. 

### 3.3. Characterization of the Spheroids on G0 Topographic Surfaces

To understand the composition of the spheroids that form only on G0, but not on the other generations of topographies, the cells were analyzed by immunofluorescence. The cells were stained for α-SMA, a biomarker for activated fibroblasts such as myofibroblasts and CAFs, DAPI for the nucleus, and alpha-fetoprotein (AFP) for tumor cells.

Figure 3 shows two focal planes of the spheroid. A magnified view of the focal plane of a spheroid on G0*Hex* with an immunofluorescence staining for α-SMA (red), DAPI (blue), and AFP (green) is presented in Figure 3a. It shows CAF cells (red) enwrapping a spheroid of tumor cells (green), positive for AFP. In Figure 3b, the focal plane is that of the topographic structures, which are visible as regular hexagonal patterns with red dots. Figure 3b shows the merged image from the red channel (Figure 3b1; SMA), the DAPI channel (Figure 3b2), and the AFP channel (Figure 3b3). It confirms that the 2D cell layer consists exclusively of CAFs and spheroid only of tumor cells. The signal for DAPI in the cells in 2D is low because of the strong signal of accumulated nuclei in the tumor spheroid. 

A reconstruction of a Z-stacks imaged by confocal microscopy of a spheroid in Figure 3c. The x-z-view (Figure 3c1) confirms the spheroid. Interestingly, it appears that the spheroid bottom is 15 μm from the level of the pyramid tips (calculated from the z-step size and number of images). This is surprising as the pyramids are only 4 µm high. A hypothesis was that during the formation of the spheroids, the cells digested the amorphous silica of the topography. Therefore, the cells were chemically removed by piranha treatment; SEM images show that cells did not degrade the SiO_2_ structures, and the topography remained intact (Figure S6). Some pyramids were found to be damaged, but this was due to mechanical damage. Therefore, we hypothesized that a change in refraction index, i.e., cell cluster, liquid, and substrate, might induce light reflection, causing an optical distortion. 

### 3.4. Tumor Cells in CAF Isolations

As the spheroids were AFP-positive tumor cells, fresh CAF isolates from 3 HCC patients were analyzed by fluorescence-activated cell sorting (FACS) for contaminating non-CAF cells (Table 1). 

In the three CAF isolates from HCC derived from patient 1–3 cancer stem cells (CSC) positive for CD13 [48,49], CD44, CD90 [49], CD133 [50], OV6 [49], epithelial cells or CSCs positive for EpCAM [49], or general tumor cells positive AFP [51] were identified by immunofluorescence. This confirmed that the CAF isolates still contained different amounts of contaminating cells, especially varying concentrations of CSC. 

**Figure 4:**
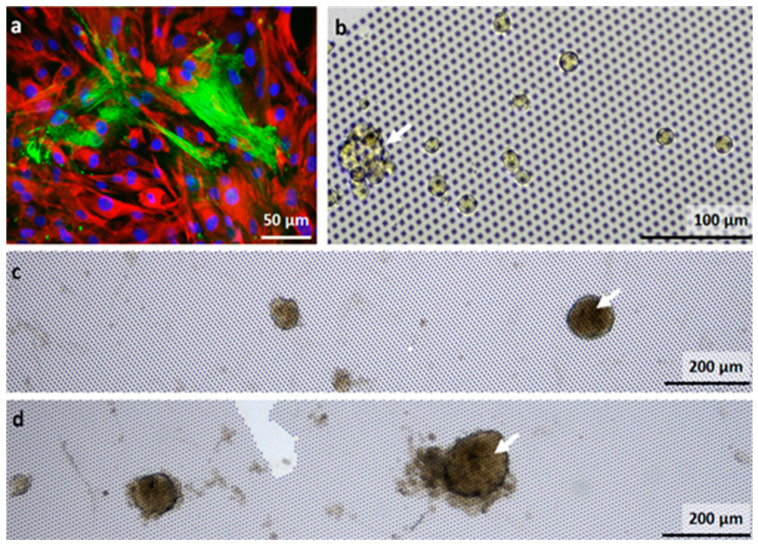
(**a**) First passage in 2D cell culture of an isolate of CAFs from primary HCC at the stained with antibodies for Vimentin (red), a cytoskeleton marker, and α-SMA (green), a marker for activated fibroblasts. The nuclei were stained with DAPI (blue); (**b**–**d**) CAFs and contaminating cells isolated from HCC of three patients (P1–3) and cultured for 6 days on G0*Hex*. White arrows indicate small cell clusters for P1 (**b**) and spheroids for P2 and P3 (**c**,**d**).

Next, the CAF isolates with the contaminating cells were cultured for 6 days on G0*Hex* topographic surfaces to correlate the microtumor/spheroid formation to the content of AFP-positive cells. For comparison, the first passage of a CAF isolate was also grown in 2D and stained for vimentin (red), a cytoskeleton marker to visualize all cells, and α-SMA (green) for the CAFs. The immunofluorescence image in Figure 4a confirms that only 20% of the cells were CAFs. 

Spheroids were found for the CAF isolates from patients P2 and P3 (Figure 4c,d), while the culture from P1 did not show spheroid formation (Figure 4b). For P1-3, 6–10 images for two substrates G0*Hex* were analyzed on days 4, 6, and 12. On day 4 for P1, 3.5 ± 2 cell clusters were found per substrate. For P2 and P3, per image 14.5 ± 2 and 12.5 ± 2 cell clusters were observed, respectively. On days 6 and 12, some of the clusters were lost due to disconnection to the surface or cell death. On day 6, for P1-3, 0.5 ± 0.7, 11.5 ± 5, and 8 ± 7 spheroids were still counted. On day 12, for P1, no cell cluster was found, while for P2, it was 3.5 ± 0.7 and for P3, 3.5 ± 2. 

The observation that most cell cluster/spheroids were detected for P2 and P3 is interesting, as the amount of AFP-positive was the highest for P1 (Table 1). There was no obvious correlation between the composition of the contaminating cells. There might be a correlation to the amount of CSC, which then differentiate in APF-positive HCC cells [52]. A full understanding of the origin of the spheroids needs more detailed studies. 

### 3.5. The Identification of 2D or 3D on Topographic Surfaces—Which Is It?

**Figure 5:**
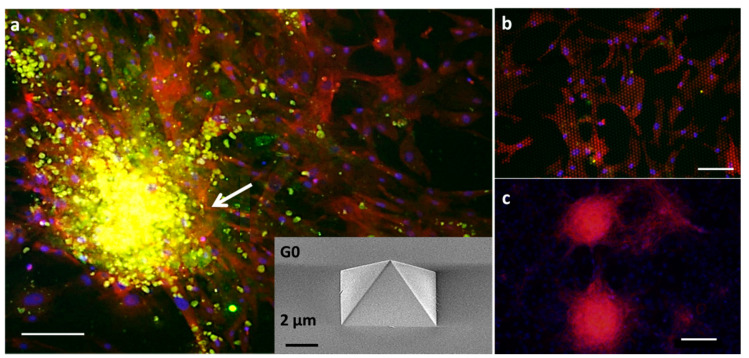
A piece of primary tumor (**a**) or peri-tumoral (non-tumor) (**b**) tissue was placed on the topographic surface G0 for 32 days. After removal of the tissue piece, the remaining cells were stained for α-SMA (red), AFP (green), and the nucleus (DAPI, blue): (**a**) merging two images to show the tumor cell distribution distance from the contact area of tumor and surface. The residual cells of the tumor tissue on G0*Sqr* consist of a mixture of tumor cells (yellow); co-localization of α-SMA (red) and AFP (green) and CAF (red) mainly localized in the contact area of tumor and surface (arrow); (**b**) the residual cells from the peri-tumoral tissue on G0*Hex* consist only of fibroblasts and invading the structured surface. They have a low red SMA signal, as they are normal fibroblasts; (**c**) the HLF cells form two dense spheroids stained by TRITC–phalloidin (red), as they do not produce AFP. Scale bar: 100 μm.

Thus far, the 2D cell layer was identified to consist of CAFs, while the spheroid consisted mainly of cancer cells. In order to confirm these observations further, pieces of tumor (Figure 5a) and peri-tumoral tissue (Figure 5b) that did not contain tumor (stem) cells were placed on G0 for 32 days, then removed, and the attached cells were stained for AFP (green) and α-SMA (red). For comparison, cells from the hepatoma cell line, HLF, and the human colorectal adenocarcinoma cell line, Caco-2, were grown on G0*Hex* for 8 days until they formed dense cell clusters (Figure 5c). HLF resemble fibroblasts in morphology but have also epithelial characteristics [53]. As this cell line does not produce AFP [53], the cells were stained with TRITC–phalloidin for actin (red, Figure 5c).

As expected, the residual cells from the tumor piece consisted of AFP-positive cells, which were localized with the highest concentration to the contact area of the tumor and topographic surface (arrow in Figure 5a). The spot-like appearance of the yellow AFP/α-SMA signal indicated that it stemmed from tumor cell invadosomes, which are known to be enriched in actin and penetrating the microenvironment [54]. Farther from the contact area, the cell layer consisted mainly of invading CAFs. If the peri-tumoral piece was removed, the cell layer consisted exclusively of fibroblast with a low signal of α-SMAm, which is an indicator for modified fibroblasts [1,55]. 

### 3.6. Applications of the Topographic Surfaces

#### 3.6.1. One-Step CAF Purification 

The difference in growth behavior of CAFs and tumor cells can be used to separate CAFs from tumors for in-depth study in a simple procedure. Usually, the CAFs isolation is a length procedure in which the isolate will undergo several passages to purify CAFs.

**Figure 6:**
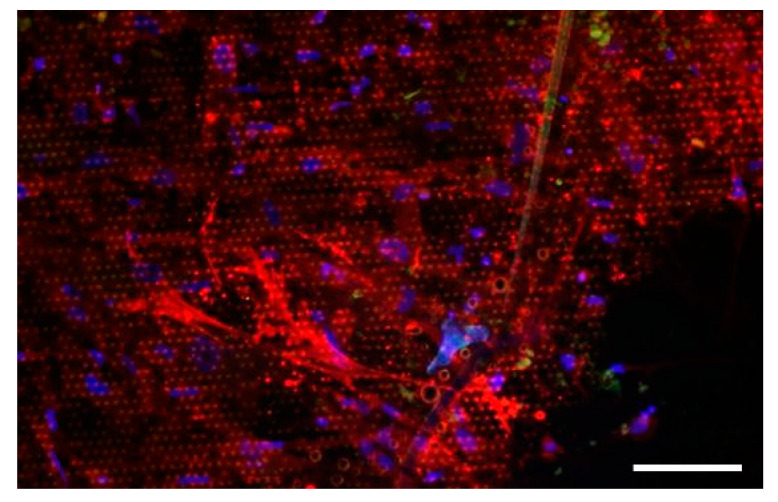
A tumor piece was placed on G1*Hex* for 32 days, and after removal, the residual cells were stained for α-SMA (red), AFP (green), and the nucleus (DAPI, blue). No signal for AFP was observed, indicating that cells were exclusively CAFs. Scale bar: 100 μm.

Considering our initial observation that the primary tumor/CAF isolate formed spheroids only on G0 but on G1 and higher generations, only 2D cell growth was observed; thus, we placed a hepatocarcinoma piece similar to the one used for Figure 5a on G1*Hex* for 32 days (Figure 6). 

It was observed that the CAFs from the primary tumor started to invade the topographic surfaces at day 2, while after 32 days, a pure CAF layer with high confluence was observed (Figure 6). This was possible because the highly motile CAFs migrated into the topographic structures and were maintained when the tumor piece was removed. Tumor cells were excluded and did not migrate in G1 or higher generations.

#### 3.6.2. Fast Attached Spheroid Formation from Cell Lines

**Figure 7:**
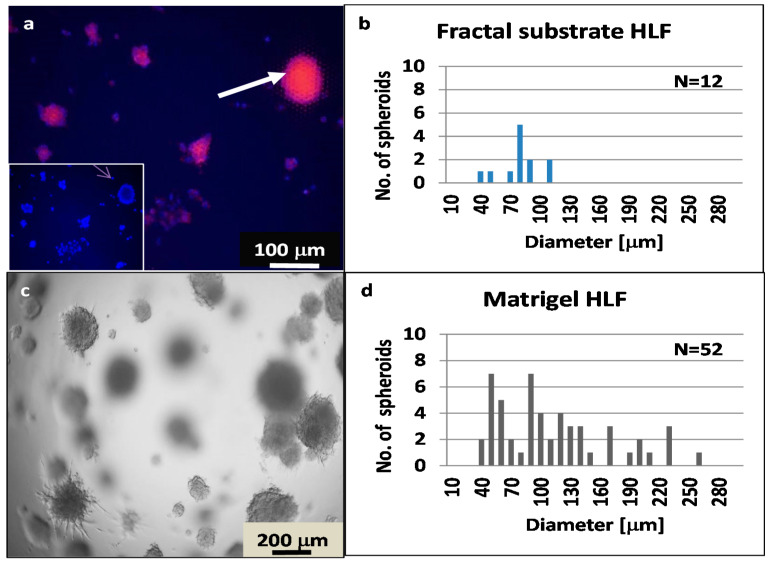
(**a**) Epifluorescence image of HLF spheroids grown on G0*Hex* for four days. The spheroids appear violet because the DAPI (blue) signal overlaps with TRITC–phalloidin staining (red) of the actin filaments. The inset shows the blue DAPI signal; (**b**) diagram of the size distribution of the spheroids on G0 as determined by image analysis with ImageJ (N = 12); (**c**) transmission image of HLF spheroids embedded in Matrigel after 13 days; (**d**) diagram of the spheroid size distribution in Matrigel, as determined by image analysis with ImageJ (N = 52).

We compared the formation of spheroids from the HLF cell line in MatriGel and on the topographic surfaces (Figure 7).

A comparison between HLF cells on G0*Hex* and in Matrigel (Figure 5c) showed that in Matrigel, the cells needed 13 days to form spheroids of around 100 µm, while they needed 4 days on G0. The average diameter of HLF spheroids on the topographic surfaces was 74 ± 20 μm (N = 12) (Figure 5a), and in Matrigel, 108 ± 57 μm (N = 52) at day 13 (Figure 7d). However, a direct comparison of both culture methods was hampered because the spheroid grew in Matrigel from embedded single cells [56], while the cells on the topographic surfaces started to cluster immediately on day 1, as seen in Figure S3.

## 4. Discussion

In this study, micro(nano)structured surfaces were used to induce 3D cell growth. The composition of our topographic surfaces was amorphous SiO_2_ but differed in the surface features (Figure 1). The number of contact points for the cells to attach to and the free space between the features varied. However, it was shown that pyramidal structures (G0) induced 2D/3D complex spheroid formation. Higher hierarchies induced the formation of 2D cell layers and the exclusion of cancer cells. These features are known to influence cell differentiation, e.g., in stem cells [57]. To the best of our knowledge, no topographical surfaces (e.g., G0) are known to promote spheroid formation by attachment. It is known that topographical surfaces can induce physical stimuli to cells via mechanoreceptors [58], i.e., laminin–integrin receptors for CAFs or other integrins in focal adhesion regions of motile cells [59,60,61,62]. This cellular behavior depends on material stiffness and topography. Stiffness is defined by elasticity, i.e., Young’s Modulus to which cells are quite sensitive [58,59,60]. This also includes material properties and cell environment [63]. 

Only a few studies work with microstructured inorganic or organic surfaces for cells growth. Araujo et al. [64] reported on templates consisting of SU-8, an epoxy-based material hexagonal with a periodic pattern of microcavities with a size ranging from 12 to 560 μm and its influence on cell growth of Chinese hamster ovary (CHO) cells. The honeycomb-like structures found the highest cell density of single, separated cells for the 80 μm cavities. However, the authors did not observe any 3D growth, and in most cavities, the growth rate and cell density were significantly lower than on flat surfaces. The study of Boccafoschi et al. [65] addressed the influence of microstructuring on myofibroblasts. In this study, the authors fabricated a square pattern of periodic ellipsoid micropillars from polydimethylsiloxane (PDMS), with a distance of 25 μm and two different heights (4 and 10 μm). These pillars were either used without further treatment or silanized to increase the hydrophobicity of the surface. After 72 h on the control PDMS flat surface, they observed patchy cell clusters (low cell numbers), while on both pillared surfaces, a continuous 2D cell sheet with a slightly higher cell density on hydrophobized pillars with contact angles (CA) higher than 90o. The fibroblasts on pillars had an increased expression of vinculin and FAK indicators for fibroblast activation. They also studied the formation of podosomes and filopodia, especially the hydrophobized pillars. Their data are in good agreement with our observation of the formation of actin-rich podosomes and filopodia of fibroblast cells interacting with the tips of the G0, as well as unidirectional bundles of actin filaments (Figure 7b or Figure S4). Other systems with silicon composition, such as those reported by Decuzzi et al. [66,67], were revealed in their works on inorganic Si-wafer with an increased random nano-roughness, which indicated that roughness enhances the proliferation of tested cell lines up to 7 days when cells reach confluence. However, they observed 2D cell growth.

Tumor spheroids, especially from the tumor pieces placed on the fractal G0 templates (Figure 3), were enwrapped by a layer of α-SMA positive CAFs, which is very similar to the distribution of CAFs in natural HCC and cholangiocarcinoma [44,68]. The α-SMA-positive CAFs separate different tumor clusters, presenting a sheath-like physical barrier for treatment and indicating poor prognosis [67]. The role of α-SMA-positive CAFs in the progression of HCC and its mutual influence on each other is supported by strong scientific evidence [16,43,69,70,71,72,73,74]. In order to understand the crosstalk of both components—tumors and CAFs—it is also important to have pure cultures of primary CAFs. 

## 5. Conclusions

An inorganic platform for the culture of 2D/3D tissue was presented. The cell culture platform consisted of a surface decorated with structures. Several structural features were tested, ranging from pyramids over octahedrons to fractals, increasing in complexity, but only features such as pyramids promoted the formation of 2D/3D clusters in a short time. The topographic structures introduced in this work showed that the cell culture of both primary cells and cell lines on these surfaces was decorated with pyramidal features, making 3D cell culture as easy as standard 2D cell culture. Furthermore, it was shown that topographical designs could also be used for cell purification.

## 6. Patents

The following patent was filed resulting from the work reported in this manuscript: PCT/NL2021/050409.

## Figures and Tables

**Table 1:** Analysis of FACS results for epithelial, stemness, and mesenchymal markers of CAFs isolates from HCC primary tumors of three patients (P1, P2, P3).

	Patient 1 (P1)			Patient 2 (P2)			Patient 3 (P3)		
		MFI			MFI			MFI	
	POS ^1^	A ^2^	G ^3^	POS	A	G	POS	A	G
AFP	13.0	2.50	1.21	50.7	3.00	1.69	2.4	0.00	0.70
CD13	52.0	1.01	0.54	65.8	4.54	4.83	0.5	0.08	0.04
CD44	79.7	12.4	9.47	25.9	5.97	2.66	89.1	10.8	13.3
CD90	76.2	7.52	5.92	34.8	20.5	5.99	83.3	31.9	26.4
CD133	−0.3	−0.02	−0.01	33.8	0.63	0.85	0.0	0.04	0.05
CD151	69.1	4.10	5.38	12.7	3.27	2.51	41.9	7.69	11.9
EpCAM	62.8	1.94	2.05	3.5	0.56	0.59	−0.2	−0.04	−0.09
OV6	0.5	−0.07	−0.35	0.6	0.70	0.14	4.1	1.64	0.51

^1^ Percentage of positive (POS, %POS) cells and/or mean fluorescence intensity (MFI) of antibody-stained cell populations (MFI, expressed as ^2^ arithmetic mean (A-mean) and ^3^ geometric mean (G-mean)) are reported. Percentages and fluorescence values are normalized to control/ isotype-related signals.

## Supplementary Materials

The following are available online at www.mdpi.com/article/10.3390/nano11123233/s1, Figure S1: Overview image of the cell distribution on substrates. The higher cell density is due to the pre-incubation of a droplet of cell suspension, Figure S2: Topographical surfaces with the respective dimensions for each geometry, Figure S3: (a) Light microscopy of CAF cells at day 1 and (b) tumor spheroids on CAF cells after day 6 of culture on G0*Sqr*, Figure S4: CAF cells 8 days after seeding on square-oriented inorganic topographic surfaces, Figure S5: The fluorescence image shows CAFs at 8 days after seeding on square-oriented inorganic surfaces decorated with structures G2*Hex*, Figure S6: SEM image of the substrate after piranha treatment for 20 min, Figure S7: Fluorescence-activated cell sorting (FACS) from different patient biopsies, Table S1: Geometrical base (b), end-octahedral diameter (B), structure height (H), and structure-to-structure nearest neighbor interspace (i) of the 3D geometries on the substrate configured either in hexagonal (*Hex*) or square (*Sqr*) lattice are listed.
