# Supplementary material for: Complex Tumor Spheroid Formation and One-Step Cancer-Associated Fibroblasts Purification from Hepatocellular Carcinoma Tissue Promoted by Inorganic Surface Topography
Source: Nanomaterials (Basel). 2021 Nov 28;11(12):3233. doi: 10.3390/nano11123233 (PMC8706479; doi:10.3390/nano11123233)
Supplement: Supplementary file 1 [file nanomaterials-11-03233-s001.zip › nanomaterials-1450163-SI.pdf]

nanomaterials

Supplementary Materials

Complex Tumor Spheroid Formation and One-Step Cancer-Associated Fibroblasts Purification from Hepatocellular Carcinoma Tissue Promoted by Inorganic Surface Topography

Francesco Dituri [1], Matteo Centonze [1], Erwin J.W. Berenschot [2], Niels R. Tas [2], Arturo Susarrey-Arce [2,*] and Silke Krol [1,*]

[1] Laboratory for Personalized Medicine, National Institute of Gastroenterology, "S. de Bellis" Research Hospital, Castellana Grotte via Turi 27, 70013 Bari, Italy; Francesco.dituri@irccsdebellis.it (F.D.); matteo.centonze@irccsdebellis.it (M.C.)
[2] Mesoscale Chemical Systems, MESA+ Institute, University of Twente, P.O. Box 217, 7500AE Enschede, The Netherlands; j.w.berenschot@utwente.nl (E.J.W.B.); n.r.tas@utwente.nl (N.R.T.)
* Correspondence: a.susarreyarce@utwente.nl (A.S.-A.); silke.krol@aol.com (S.K.)

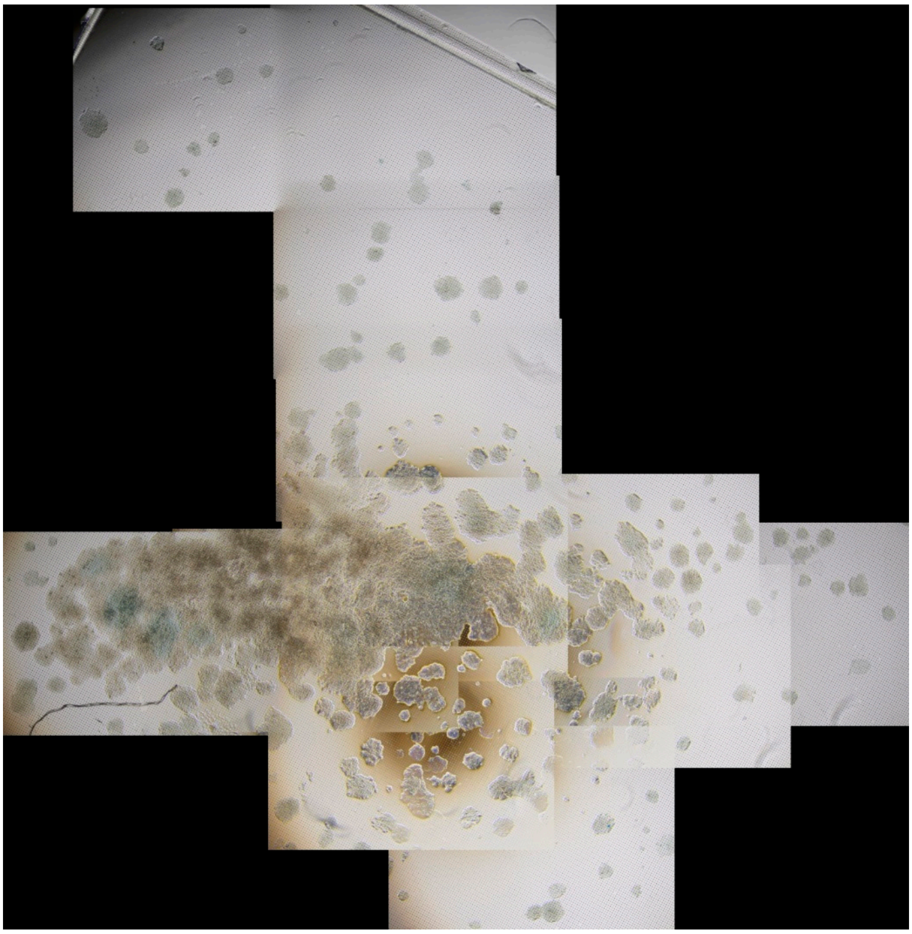

Figure S1. Overview image of the cell distribution on topographic substrates. The higher cell density is due to the pre-incubation of a droplet of cell suspension before filling the well with medium.

**G0**	
Sqr<br>*Hex*<br>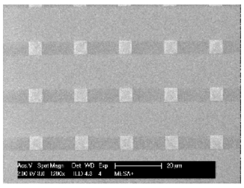<br>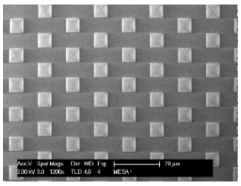<br>*b* (um) 5.7<br>6.0	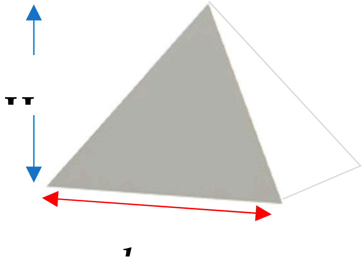
**G1**	
Sqr<br>*Hex*<br>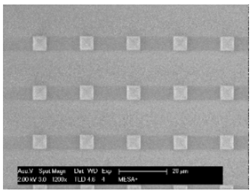<br>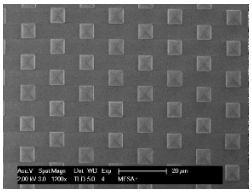<br>*b* (um) 5.8<br>6.1<br>*1/2b* (um) 5.0<br>5.8	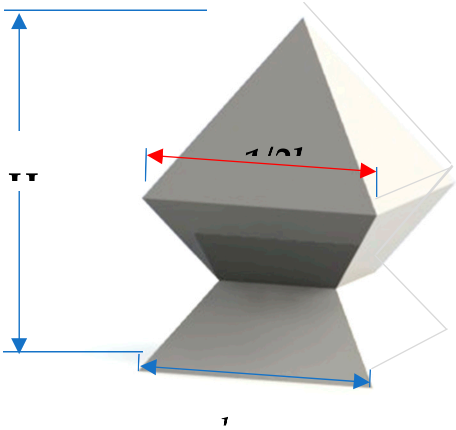
**G2**	

Sqr<br>*Hex*<br>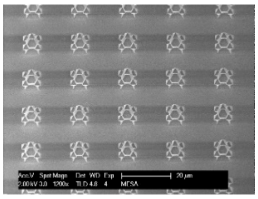<br>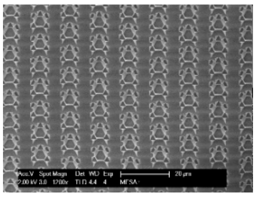<br>*b* (um) 5.8<br>6.2<br>*1/2b* (um) 4.9<br>5.5<br>*b1* (um) 2.5<br>2.5	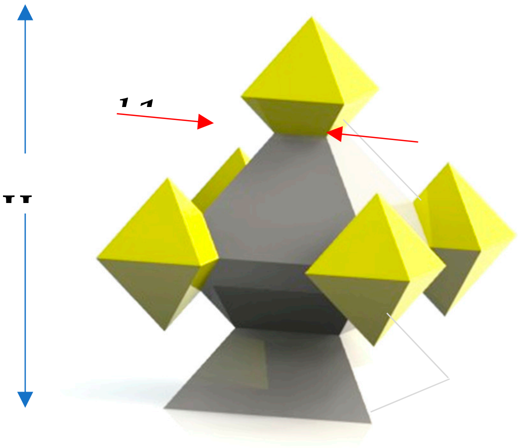
**G3**	
Sqr<br>*Hex*<br>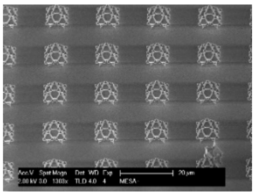<br>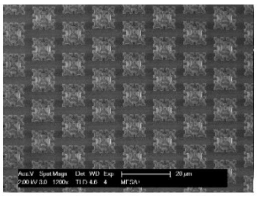<br>*b* (um) 6.0<br>6.3<br>*1/2b* (um) 4.8<br>5.2<br>b1 (um) 3.1<br>3.0	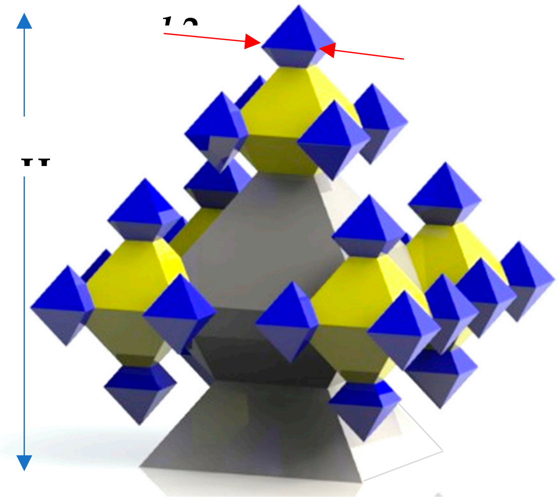

b2 (um) 1.2<br>1.2	
**G4**	
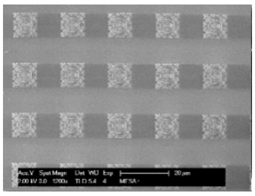<br>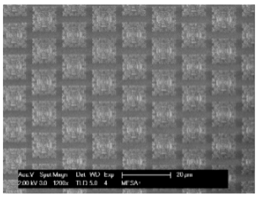<br>B (um) 5.8<br>6.2<br>1/2b (um) 5.3<br>5.2<br>*b1* (um) 3.2<br>3.1<br>*b2* (um) 1.4<br>1.2<br>*b3* (um) 0.6<br>0.6	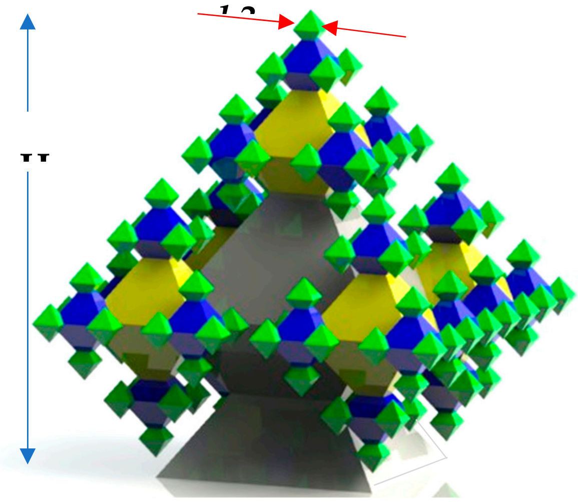

Figure S2. Topographic surfaces with the respective dimensions for each geometry.

Table S1. Geometrical base (***b***), end-octahedral diameter (***B***), structure height (***H***), and structure-to-structure nearest neighbor interspace (***i***) of the 3D-geometries on the substrate configured either in hexagonal (***Hex***) or square (***Sqr***) lattice are listed.

G	***b, B*** [a)] ***[µm]***	***H*** [b)] ***[µm]***	***I*** [a)] ***[µm]***
		Sqr	
**G0*Sqr***	5.7, -	4.03	20.0
**G1*Sqr***	5.8, 5.0	8.10	19.9
**G2*Sqr***	5.8, 2.5	10.0	14.9
**G3*Sqr***	6.0, 1.2	11.4	12.5
**G4*Sqr***	5.8, 0.6	11.4	11.3
		Hex	
**G0*Hex***	6.0, -	4.2	12
**G1*Hex***	6.1, 5.8	8.0	11.9
**G2*Hex***	6.2, 2.5	10.2	6.9
**G3*Hex***	6.3, 1.2	11.3	4.5
**G4*Hex***	6.2, 0.6	11.3	3.3

[a)]Structure base, end-octahedral diameter, and structure-to-structure nearest neighbor interspace are measured from SEM images. [b)]Height was estimated using trigonometric analysis. For each structure dimension, see Figure S2.

Fabrication of the topographic surfaces

The fabrication step consists of depositing and patterning a hard oxide mask on a silicon wafer. Using anisotropic etching (25%KOH@ 75 °C) inverted pyramids are formed. After RCA-2 cleaning, a selective etching step strips the SiO_2 hard mask (1%HF), and the G0 mold is ready.G1–G3 are formed by a repetitive sequence of processing steps on top of substrate G0, which consist of dry oxidation @ 1100 °C with the formation of a conformal oxide formation over the silicon mold containing the topographic features. The SiO_2 is conformally deposited, excepting at the sharp concave features of the mold, i.e., the intersections of 2, 3, and 4 planes are present on the silicon wafer in the form of ribs, 3 plane apices, and 4 plane apices resp, which have a reduced thickness of SiO_2 because of stress-induced oxidation retardation. The concave features with the thinnest oxide thickness (e.g., 3plane/4plane apices) can be selectively opened using timed isotropic etching (1%HF). Through these openings, octahedral cavities bounded by Si(111) planes will form during a subsequent timed anisotropic etching (25%TMAH@70°C) step. This step determines the size of the octahedral features. G1 is tuned so that the largest width equals the size of the base of the mold G0. By repeating the sequence described above and reducing the etching time by half compared to the previous step, the feature size will decrease by a factor 2, while the number of octahedral features increases by factor 5. In this way, G2 and G3 are formed [1,2]. In the case of G4, the final iteration on top of G3 is, instead of relying on oxidation retardation, performed by the following processing steps: silicon nitride corner lithography, local oxidation of silicon, selective stripping of the remaining silicon nitride. The next step is the same as before: timed anisotropic etching (25%TMAH@70 °C) step [1,2]. After creating all G0-G4 mold wafers, a structural SiO_2 thin film is grown, and these wafers are anodically bonded to borosilicate glass wafers. By pre-dicing in 1cm^2 pieces and dissolving the silicon mold wafer in the anisotropic etchant with high selectivity against SiO_2 (25%TMAH@70 °C), the 1cm^2 topographic substrates are ready to use [1,2].

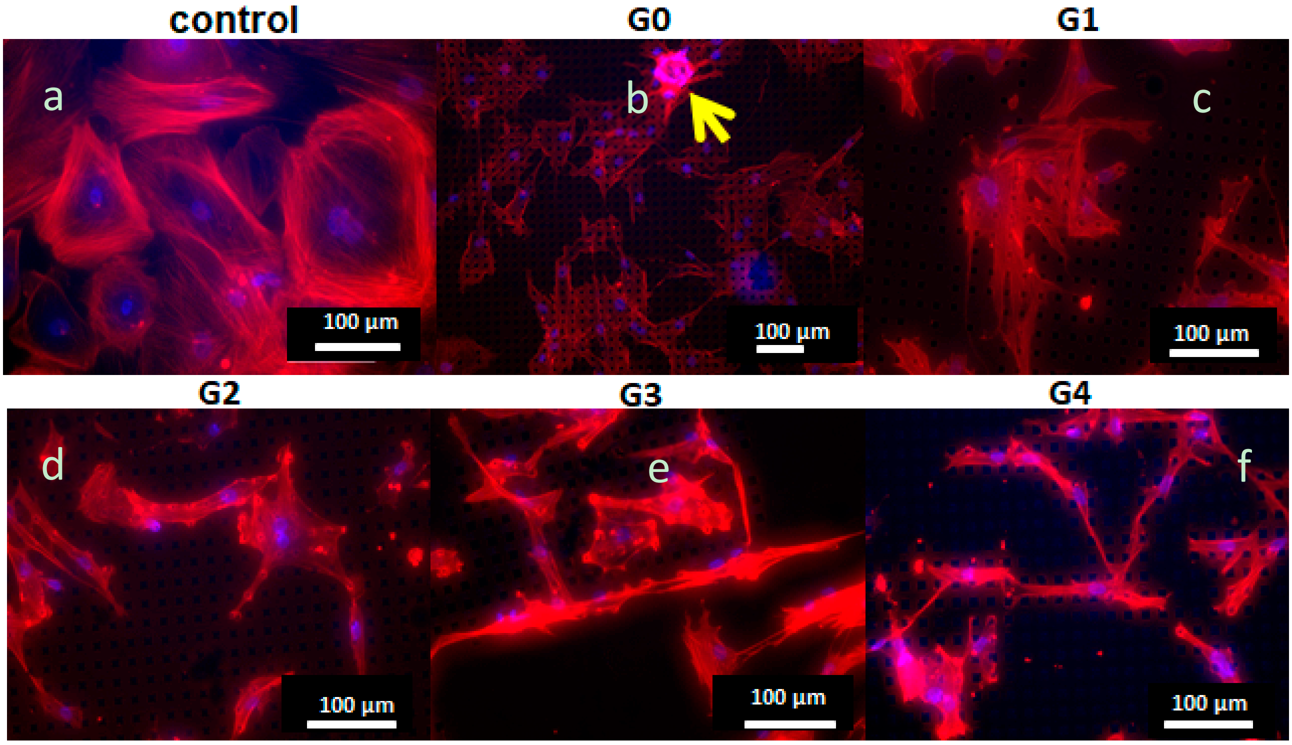

Figure S3. CAF cells 8 days after seeding on *Sqr*-oriented inorganic topographic surfaces. The nuclei are stained by DAPI (blue) and the actin filaments by TRITC-phalloidin (red). The yellow arrow indicate a spheroid on G0. Scale bar: 100 µm.

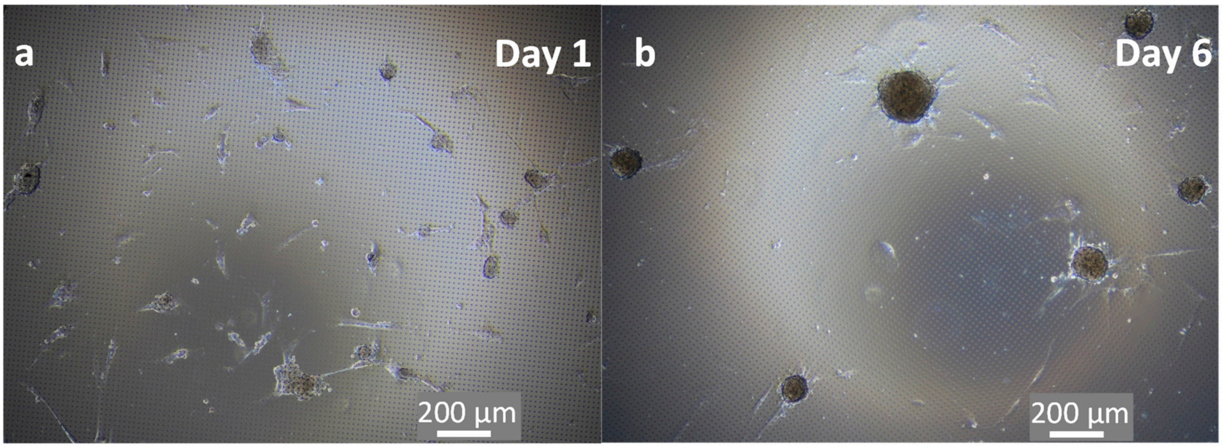

Figure S4. (**a**) Light microscopy of CAF cells at day 1, and (**b**) tumour spheroids on CAF cells after day 6 of culture on G0*Sqr*. Scale bar: 200 µm.

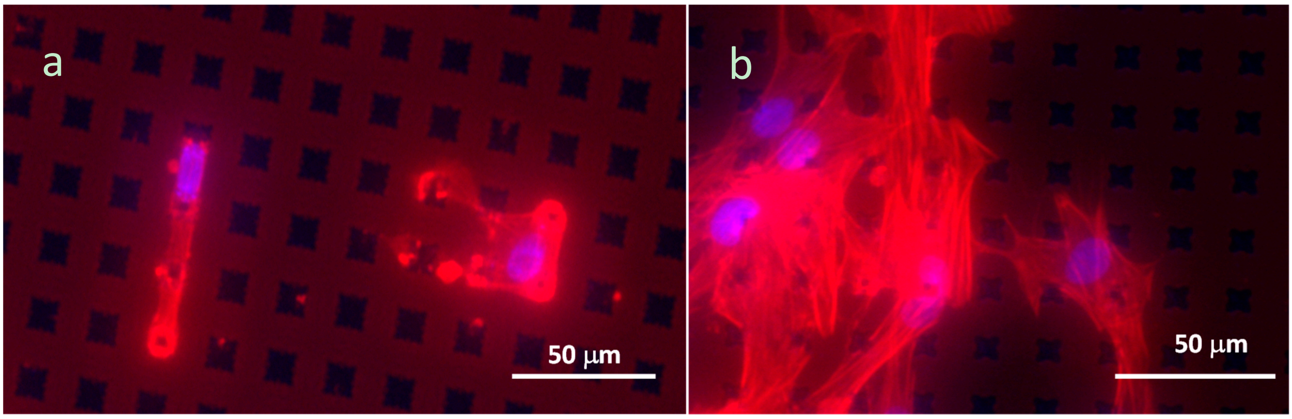

Figure S5. The fluorescence image shows CAFs at 8 days after seeding on Sqr-oriented inorganic surfaces decorated with structures G2*Hex*. The nuclei are stained by DAPI (blue) and the actin filaments by TRITC-phalloidin (red). Scale bar: 50 μm.

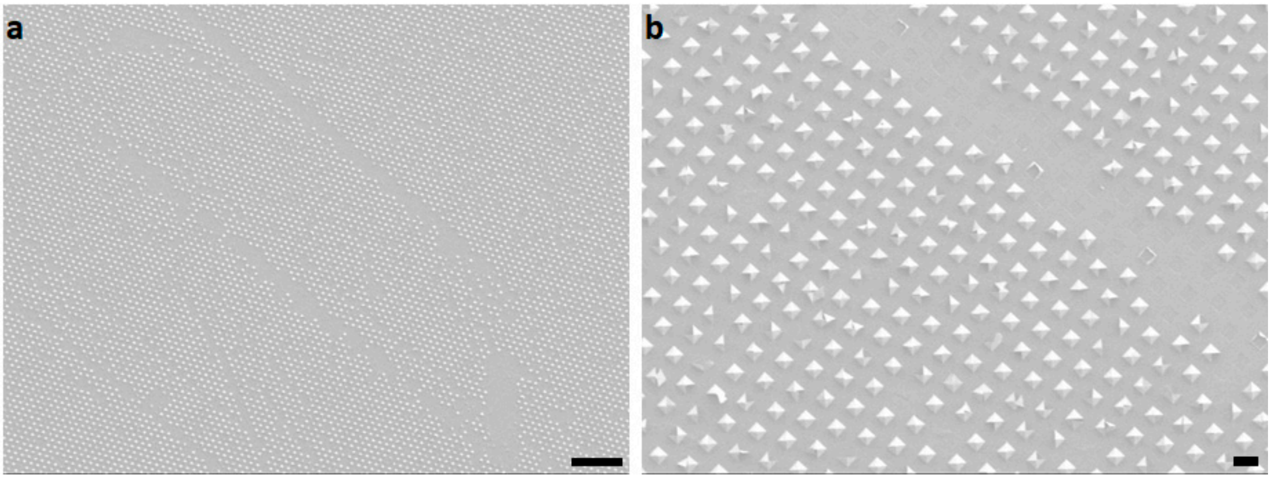

Figure S6. SEM image of the substrate after piranha treatment for 20 min. The integrity of the pyramids remains similar to the start of the experiments. Scale bar (a): 100 µm. Scale bar (b): 10 µ

nanomaterials MDPI

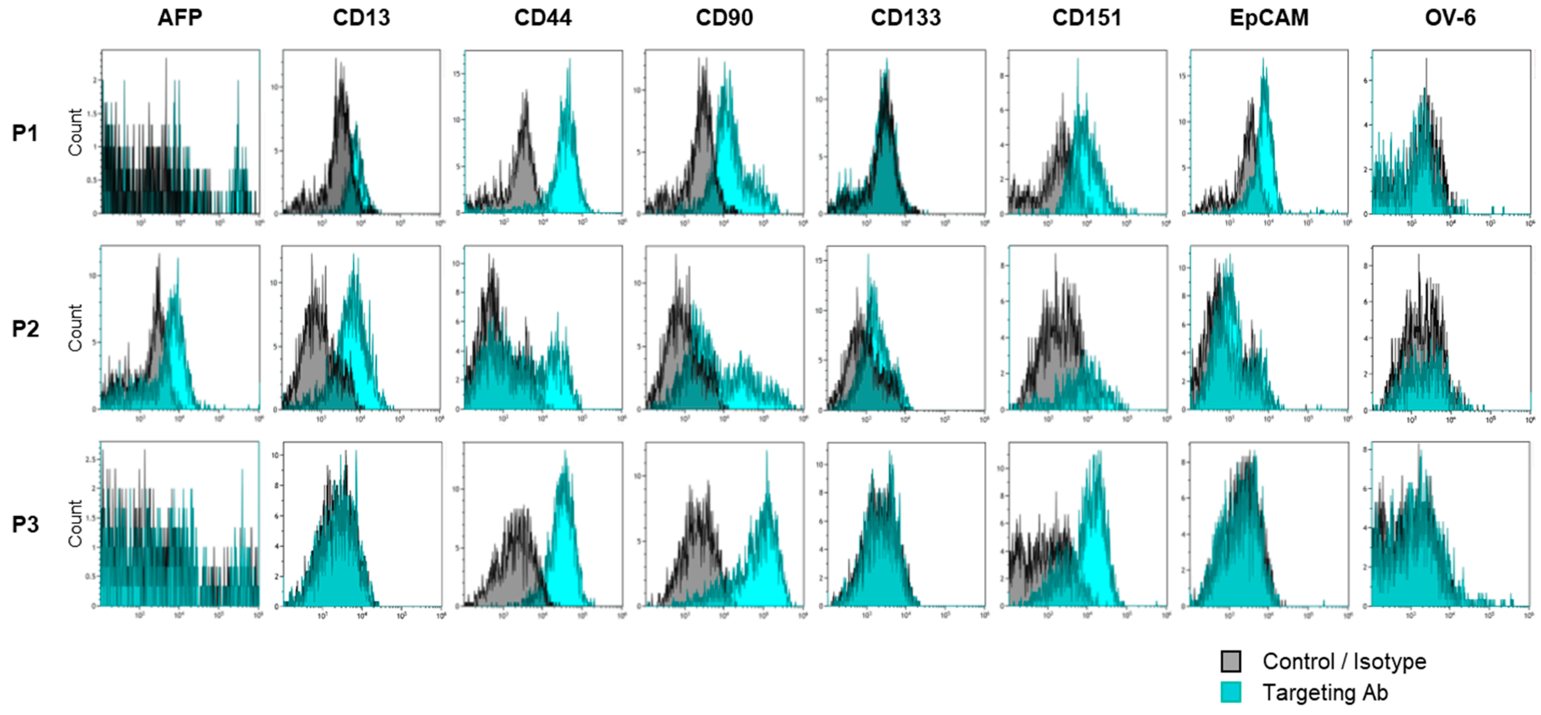

Figure S7. Flow cytometry analysis of CAFs from different patient biopsies. The results of the analysis are summarized in **Table 1** in the main text.

References

1. Berenschot, E.J.W.; Jansen, H.V.; Tas, N.R. Fabrication of 3D fractal structures using nanoscale anisotropic etching of single crystalline silicon. *J. Micromech. Microeng*. **2013**, *23*, 055024
2. Berenschot, J.W.; Tiggelaar, R.M.; Geerlings, J.; Gardeniers, J.G.E.; Tas, N.R.; Malankowska, M.; Pina, M.P.; Mallada, R. 3D-fractal engineering based on oxide-only corner lithography. In Proceedings of the 2016 Symposium on Design, Test, Integration and Packaging of MEMS/MOEMS, DTIP 2016, Budapest, Hungary, 30 May–2 June 2016.
